# Debt and Death

**Published:** 1878-11

**Authors:** 


### DEBT AND DEATH.

General Jackson once said that any man who traded on a borrowed capital ought to break; be that as it may, I consider him a radically dishonest man who embarks in business wholly on a borrowed capital, because he is willing to endanger his friend for the chance of his own profit; he cannot lose, but his friend may. James Harper, one of the best and purest men I ever knew, a Virginia gentleman, of the old school, whose heart was welling up unceasingly with human kindness to all around him, once said to a gentleman who counted his fortune by hundreds of thousands, and who voluntarily offered to be drawn upon for any amount, replied: "*I can't consent to make a fortune at the risk of my friend.*" A mercantile gentleman, who was honor personified, *Joseph Stephens*, once said to me: "I leave my bed of a morning bathed in perspiration, in the agony of device for meeting the engagements of the day." We all know that the fear of not being able to meet pecuniary engagements is a frequent cause of insanity and suicide to men of refinement and of a high sense of honor, while thousands are wasting away around us under the harassing pressure of debt. The temper is uneven; at one time sad, at another almost unendurably irritable; the appetite is variable, if any at all; the nights are restless, the sleep unrefreshing; gladness hies from home, and silent gloom pervades the fireside circle, thus verifying the scripture assertion that *they who hasten to be rich shall pierce themselves through with many sorrows.*

In view then of its health-destroying influences, I may very properly give the admonition in this journal, *avoid debt:* shun it as you would the pestilence that walketh in darkness, or the plague that wasteth at noonday; consider it your mortal enemy—the enemy of your body, your health, your happiness, your soul—the enemy of your wife, your children, and every kindred tie.

Take almost any business man, and he will tell you in more than three cases out of four, that he has lost more by bad debts than he is now worth. It is a monstrous fallacy that "if a man expects to become rich he must go in debt." The sentiment originated in the heart of a rogue. Debt is not the policy of the most successful men.

